# *Lactobacillus agilis* SNF7 Presents Excellent Antibacteria and Anti-Inflammation Properties in Mouse Diarrhea Induced by *Escherichia coli*

**DOI:** 10.3390/ijms252413660

**Published:** 2024-12-20

**Authors:** Mingque Feng, Jia Cheng, Yalan Su, Jingdi Tong, Xiangfu Wen, Tianxiong Jin, Meiyi Ren, Deyuan Song, Jinshang Song, Xiaohan Li, Qinna Xie, Mingchao Liu

**Affiliations:** 1 College of Veterinary Medicine, Hebei Agricultural University, Baoding 071000, China; fengmingque@163.com (M.F.); chengjia@hebau.edu.cn (J.C.); 20222200586@pgs.hebau.edu.cn (Y.S.); tongjingdi@pgs.hebau.edu.cn (J.T.); 20232200610@pgs.hebau.edu.cn (X.W.); tianxiongjin@nwafu.edu.cn (T.J.); renmeiyi@psg.hebau.edu.cn (M.R.); 20232200614@pgs.hebau.edu.cn (D.S.); songjinshang@psg.hebau.edu.cn (J.S.); 20232200611@pgs.hebau.edu.cn (X.L.); 20237201621@pgs.hebau.edu.cn (Q.X.); 2 College of Veterinary Medicine, China Agricultural University, Beijing 100193, China; 3 College of Animal Science and Technology, Hebei Agricultural University, Baoding 071000, China

**Keywords:** *Lactobacillus agilis* SNF7, *Escherichia coli*, diarrhea, whole genome, probiotic properties

## Abstract

*Escherichia coli* (*E. coli*) is a common pathogen that causes diarrhea in newborns and animals. Antibiotics are typically used to treat bacterial diarrhea, a global intestinal health issue. Probiotics have gained interest as a potential substitute for antibiotics in the management of *E. coli*-induced diarrhea and present novel therapeutic options. In this study, the probiotic properties of *Lactobacillus agilis* SNF7 (*L. agilis* SNF7) isolated from feces were investigated, and whole genome sequencing was performed to evaluate the properties of the strain. Furthermore, we investigated the protective effects of *L. agilis* SNF7 in a mouse model of *E. coli* K99 infection. *L. agilis* SNF7 exhibits a high survival rate in artificial gastroenteric fluid and bile salt environments, along with an antagonistic effect against *E. coli* O_111_:K_58_ (B_4_), *Staphylococcus aureus* (*S. aureus*), and *E. coli* K99. Multiple genes with probiotic properties, including bacteriostasis, anti-inflammation, antioxidant, CAZyme, and the utilization of carbohydrate compounds, were identified in genome. *L. agilis* SNF7 prevented the gut barrier from being damaged by *E. coli* K99, reducing the clinical manifestations of the infection. Furthermore, *L. agilis* SNF7 reduced the expression of inflammatory cytokines (IL-6, IL-1β, and TNF-α) by inhibiting the phosphorylation of proteins linked to the NF-κB and MAPK signaling pathways. *L. agilis* SNF7 improved the intestinal microbial barrier, controlled the balance of the intestinal microecology, and reduced the entry of harmful microbes into the intestine. By controlling gut flora and reducing the inflammatory response, *L. agilis* SNF7 may be able to prevent and treat *E. coli* K99 infections. The application of *L. agilis* SNF7 in the creation of probiotic formulations to stop intestinal illnesses brought on by *E. coli* infections is clarified by this work.

## 1. Introduction

When administered in sufficient doses, probiotics have been established to be beneficial bacteria that are active in the body [1]. According to the reports, probiotics are crucial for enhancing health and preventing illness and bacterial overgrowth [2]. Through commensal microbes’ probiotic action, the gut microbiota plays a crucial role in preserving human intestinal homeostasis. Gut microbiota imbalance is linked to intestinal mucosal barrier damage and inflammation, or dysbiosis, which frequently affect host immunity [3,4].

In the intestines of both humans and animals, lactic acid bacteria (LAB) are a significant autochthonous microbiota [5]. They constitute key probiotics that are essential for young animals’ health in preventing infections, and the host does not establish an immune response to them in long-term co-evolution [6,7]. Additionally, isolated *Lactobacillus* strains were shown to exhibit an inhibitory impact against pathogenic organisms such as *Salmonella Typhimurium* and *E. coli* K88 in in vitro investigations [8]. *Lactobacillus salivarius* alleviated the inflammation response through reducing inflammation-related cytokines and displayed a potency in the enhancement of IPEC-J2 cell integrity [9]. Therefore, LAB have a high research value as probiotics. An essential first step in regulating the gut microbiota and overall health is the first colonization of bacteria in the human gut. One study examined the presence of LAB in newborns’ feces during the first week of life and discovered that *Lactobacillus agilis* (*L. agilis*) had colonized the neonatal gut [10]. Notably, numerous studies demonstrating *L. agilis* show strong probiotic characterization and immunomodulatory and anti-intestinal inflammatory properties [11,12,13]. *L*. *agilis* preventative and therapeutic benefits for diarrhea-causing pathogenic bacterial infections, however, have not been explored.

*Escherichia coli* (*E. coli*) is believed to be a normal part of the gut microbiota that inhabits both human and animal digestive tracts. However, the breeding business suffers significant financial losses when enteropathogenic *Escherichia coli* causes diarrhea in young animals, particularly calf and newborn piglets [14]. Diarrhea is caused by an *E. coli* infection, which modifies the integrity of tight junctions and causes intestinal dysfunction and inflammation [15]. Antibiotic-resistant bacteria are common in livestock worldwide as a result of antibiotic abuse, which also results in drug residues, a breakdown of intestinal flora, and other conditions [16]. Nowadays, infections brought on by pathogenic *E. coli* are commonly treated in clinics with antibiotics. Finding antibiotic substitutes in animal husbandry is therefore essential, and probiotics stand out among the other options as effective treatments for *E. coli* infections.

In the present study, one strain of *L. agilis* was isolated from calf feces and designated as *L. agilis* SNF7. We explored the probiotic properties and biological characteristics of the *L. agilis* SNF7 strain. Additionally, a mouse model was developed to examine how *L. agilis* SNF7 prevented diarrhea caused by *E. coli* K99. In order to theoretically support the microecological preparation to prevent *E. coli*-induced diarrhea, the purpose of this study is to assess the efficacy of *L. agilis* SNF7 in treating and preventing *E. coli* K99 in mice.

## 2. Results

### 2.1. Colony Morphology and Genetic Analysis

**Figure 1:**
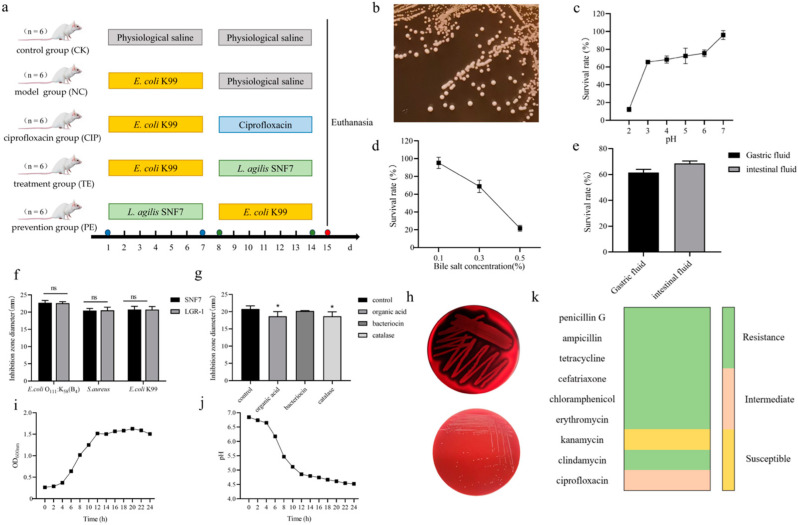
Experimental design and the characteristics of *L. agilis* SNF7 in vitro. (**a**) Experimental design; (**b**) colony morphology; (**c**) survival of *L. agilis* SNF7 at different pH levels; (**d**) survival of *L. agilis* SNF7 at different bile salt concentrations; (**e**) survival of *L. agilis* SNF7 in artificial gastrointestinal fluid; (**f**) inhibitory effect of *L. agilis* SNF7 on three strains of pathogenic bacteria; (**g**) inhibitory effect of *L. agilis* SNF7 on three strains of pathogenic bacteria after different treatments; (**h**) hemolysis of *L. agilis* SNF7 (the picture above is *S. aureus* and the one below is *L. agilis* SNF7); (**i**) growth curve of *L. agilis* SNF7; (**j**) acid production curve of *L. agilis* SNF7; (**k**) sensitivity of *L. agile* SNF7 to antibiotics. Compared with the CK group, ns means has no significant difference (*p* > 0.05), * means significant difference (*p* < 0.05).

Figure 1b shows the colonies are smooth, spherical, and milky white. After Gram staining, the microbial cells from that one colony were identified as Gram-positive bacilli. Furthermore, SNF7 was found to be 99% identical to *Lactobacillus agilis* by gene sequencing analysis.

### 2.2. Acid, Bile Salt, Gastric Fluid, and Intestinal Fluid Tolerance

For six hours, *L. agilis* SNF7 was cultured at various pH values (2, 3, 4, 5, and 6) or with bile salt (0.1%, 0.3%, and 0.5%). Strong acid tolerance was demonstrated by the *L. agilis* SNF7 strain (pH = 3; Figure 1c). The survival rate of *L. agilis* SNF7 reached 65.51%. *L. agilis* SNF7, on the other hand, demonstrated remarkable resistance to bile salt, achieving a survival rate of 68.82% (Figure 1d). In this study, *L. agilis* SNF7’s survival rate was evaluated in the simulated gastric and intestinal juice during a 4 h incubation period at 37 °C. The survival rates of *L. agilis* SNF7 were 61.58% and 68.62% with gastric and intestinal juices, respectively (Figure 1e).

### 2.3. Antibacterial Activity

The obtained results reveal that *L. agilis* SNF7 has positive antibacterial activity against *E. coli* O_111_:K_58_ (B_4_), *S. aureus*, and *E. coli* K99, with inhibition circle diameters of 22.73 ± 0.6, 19.93 ± 0.35, and 20.80 ± 0.89 mm, respectively (Figure 1f).

Different pH values have an impact on *L. agilis* SNF7’s antibacterial activity. The inhibitory action against *E. coli* K99 was considerably reduced when the supernatant was brought to a neutral condition (pH 7). Using proteinase K in the *L. agilis* SNF7 supernatants did not affect the antibacterial activity of *L. agilis* SNF7. When catalase was added to the *L. agilis* SNF7 supernatants, the inhibitory activity against *E. coli* K99 was significantly reduced in the treated supernatants compared to the untreated ones (Figure 1g) (*p* < 0.05). In this case, the production of organic acids and hydrogen peroxide may be the source of the inhibitory effect.

### 2.4. Antibiotic Susceptibility

Further research on *L. agilis* SNF7 demonstrated how susceptible this lactic acid bacterium is to several antibiotics (Figure 1k). The strain was entirely vulnerable to penicillin G, ampicillin, tetracycline, cefatriaxone, clindamycin, erythromycin, and chloramphenicol, but only moderately resistant to ciprofloxacin, and the strain was strongly resistant to kanamycin.

### 2.5. Hemolytic Activity

In this investigation, *L. agilis* SNF7 showed negative hemolysis (non-beta hemolysis) (Figure 1h).

### 2.6. The Biological Characteristics of L. agilis SNF7

The growth and acid production curves of *L. agilis* SNF7 in MRS broth at 37 °C are shown in Figure 1i,j. After inoculation, *L. agilis* SNF7 had exponential phase growth 2–12 h later and stationary phase growth 14 h later. As a result, the culture supernatant’s pH decreased significantly after 4 h and stayed rather constant after 12 h.

### 2.7. General Genome Features of L. agilis SNF7

Table 1 showed the basic information on genome of *L. agilis* SNF7.The single circular chromosome of *L. agilis* SNF7 had a G + C% composition of 41.63% and measured 2,214,422 base pairs (bps). The average length of the 2156 genes that were found was 900.23 bp. There were 24 rRNAs and 91 tRNAs on the chromosome.

### 2.8. Genomic Predictive Function of L. agilis SNF7

**Figure 2:**
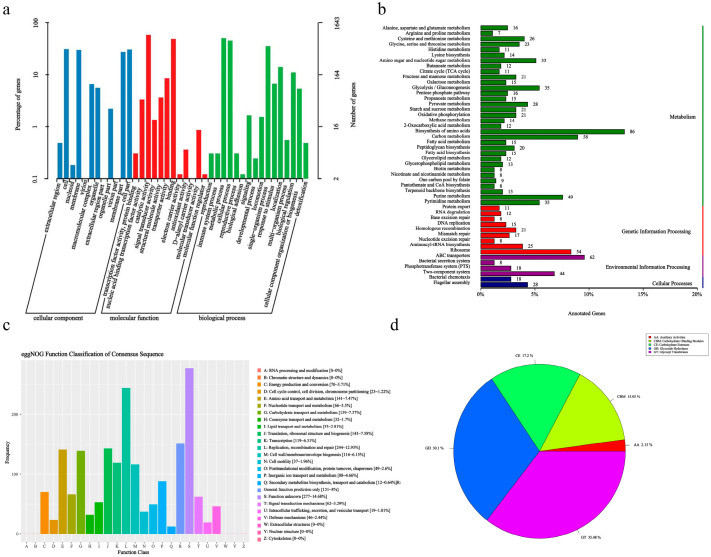
Genomic predictive function of *L. agilis* SNF7. (**a**) Annotation of the genome of *L. agilis* SNF7 in the GO database; (**b**) annotation of the genome of *L. agilis* SNF7 in the KEGG database; (**c**) annotation of the genome of *L. agilis* SNF7 in the eggNOG database; (**d**) annotation of the genome of *L. agilis* SNF7 in the CAZy database.

A total of 1643 genes were annotated in the GO classification for *L. agilis* SNF7 (Figure 2a), which can be split into three main categories: molecular function (1051, 54.0%), cellular component (372, 22.6%), and biological process (220, 13.4%). The analysis revealed that catalytic activity, binding activity, and transporter activity were the top-three most-annotated *L. agilis* SNF7 in the molecular function (MF) category; the sections on cells, membranes, cell parts, and membrane sections in the cellular component (CC) and the biological process (BP) categories had the highest annotation levels, with the highest annotation levels being found in the metabolic process, cellular process, single-organism process, and single-organism process. The sections on metabolic process, cellular process, single-organism process, localization, and biological control are highly annotated under the biological process (BP) category.

A total of 1211 genes of *L. agilis* SNF7 were functionally annotated in the KEGG database (Figure 2b), distributed in 48 pathways corresponding to four key functions: metabolism, genetic information processing, environmental information processing, and cellular processes. The majority of these genes (700) were enriched in amino acid metabolic pathways (86), carbohydrate metabolism (58), and purine metabolism (49), suggesting that *L. agilis* SNF7 may be involved in a variety of nutrient catabolism and energy metabolism processes in the host intestine. Apart from the metabolic pathways, certain genes that were encoded were associated with genetic information processing (171), environmental information processing (132), and cellular processes (46) pathways. These results imply that *L. agilis* SNF7 possesses the ability to swiftly adapt to its surroundings and maintain its survival, as well as having the capacity to perceive outside cues and effectively move materials between its internal and external environments.

Furthermore, a large number of antimicrobials, anti-inflammatory, and immunoregulatory related genes and their pathway information in the KEGG database have been annotated by *L. agilis* SNF7, which is showed in Table 2. The genome of *L. agilis* SNF7 contains genes related to metabolic processes like antibiotics and biodegradation; it also contains genes related to immunity and inflammation that can regulate immunity and inflammation, and it contains genes that can regulate pathogenic bacterial infection. *L. agilis* SNF7 is known to harbor six genes linked to antibiotic metabolism, which are involved in the biosynthesis of neomycin, streptomycin, acarbose, and valinomycin; 154 genes linked to metabolic processes, such as the synthesis and degradation of compounds related to antibacterial and anti-inflammatory properties; and 18 genes linked to the regulation of colonization by both pathogenic and self-bacteria.

Based on the eggNOG annotation (Figure 2c), a total of 1854 genes (85.99%) were annotated in the *L. agilis* SNF7 bacterium. These genes were spread over 20 COG categories. These consisted of 244 genes annotated for lipid transport and metabolism; 143 genes for translation, ribosome structure, and biogenesis; 141 genes for amino acid transport and metabolism; and 139 genes for carbohydrate transport and metabolism. Furthermore, 277 genes did not correspond to any particular activities.

Figure 2d displays the results of the CAZy database annotation. For *L. agilis* SNF7, a total of 85 genes were annotated. The glycosyltransferase (GT) gene of *L. agilis* SNF7 had the highest annotation, accounting for 35.48% of the genes related to carbohydrates; the glycoside hydrolase (GH) gene, which ranked second in the number of annotations, annotated 30.1% of the genes related to carbohydrate metabolism; the sugar esterase (CE), which catalyzes the de-esterification of different carbohydrate substrates; the carbohydrate binding module (CBM), which accounted for 15.05% of the total annotation results; and the auxiliary enzyme gene (AA), which had the least amount of annotations. According to the aforementioned findings, *L. agilis* SNF7 mostly uses glycoside hydrolases and glycosyltransferase-producing pathways to carry out the metabolism of carbohydrates.

The genome of *L. agilis* SNF7 has no plasmid elements. A total of four CRISPR repeat sequences in the genome and 11 Cas genes of 10 Cas types were detected (Table 3).

### 2.9. Safety Prediction of L. agilis SNF7

Two antibiotic resistance genes in total were assigned to *L. agilis* SNF7 from the CARD database (Table 4). They are the aminoglycoside resistance gene *APH (7″)-Ia* (GE000051) and the macrolide resistance gene *mefA* (GE000947).

### 2.10. Effects of L. agilis SNF7 on the Physiological Indexes of E. coli K99-Induced Diarrhea Mice

**Figure 3:**
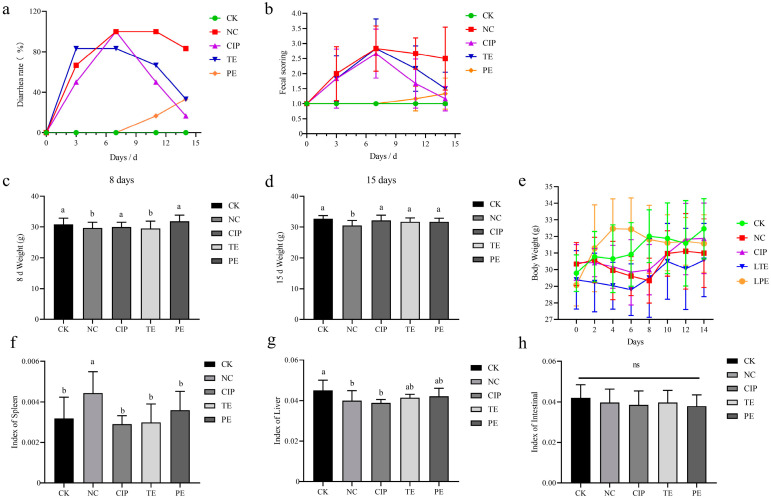
Effects of *L. agilis* SNF7 on the physiological indexes of *E. coli* K99-induced diarrhea mice. (**a**) Diarrhea rate of mice during the test period; (**b**) fecal scores of mice during the test period; (**c**) body weight of mice in each group on day 8 of the experiment; (**d**) body weight of mice in each group on day 15 of the experiment; (**e**) changes in body weight of mice during the experiment; (**f**) spleen index; (**g**) liver index; (**h**) intestinal index. Same letters on columns means the difference is not significant (*p* > 0.05), ns means has no significant difference (*p* > 0.05). The difference is significant (*p* < 0.05) when there are no identical letters between the columns.

With the exception of the CN and PE groups, all groups experienced a high rate of diarrhea on the seventh day of the experiment, according to the mice’s diarrhea rate (Figure 3a). On the fourteenth day, following the termination of *E. coli* K99, the NC group’s diarrhea rate reduced the slowest. On the seventh day of modeling, the mice in each group’s fecal index peaked (Figure 3b). Following antibiotic treatment, the mice’s fecal index dropped the most. In the meantime, the TE group’s fecal rate also dropped noticeably. Additionally, even though there were not many mice with diarrhea, the PE group’s animals experienced diarrhea on the fourteenth day following the cessation of *L. agilis* SNF7 intervention.

To find out how *L. agilis* SNF7 affected diarrhea, the mice’s body weights were measured. According to the findings, mice treated with *E. coli* K99 had significantly lower body weights than mice that were not infected (*p* < 0.05), and there was no discernible difference between the mice in the PE and CIP groups (*p* > 0.05; Figure 3c). Although there was no discernible difference between the CIP, TE, and PE groups after 15 days (*p* > 0.05), the mice in the NC group still had considerably lower body weights than those in the CN group (*p* > 0.05; Figure 3d). Following *E. coli* K99 administration, the spleen index manifested a significant enhancement (*p* > 0.05); the application of *L. agilis* SNF7 and treatment with ciprofloxacin effectively restored the condition to control levels (Figure 3e). *E. coli* K99 administration also decreased the liver index (*p* > 0.05), although prevention and treatment did not significantly restore these changes (Figure 3f). But, none of the groups exhibited significant changes in the intestinal index (*p* < 0.05; Figure 3g).

### 2.11. Effects of L. agilis SNF7 on Intestinal Physical Barriers of E. coli K99-Induced Diarrhea Mice

**Figure 4:**
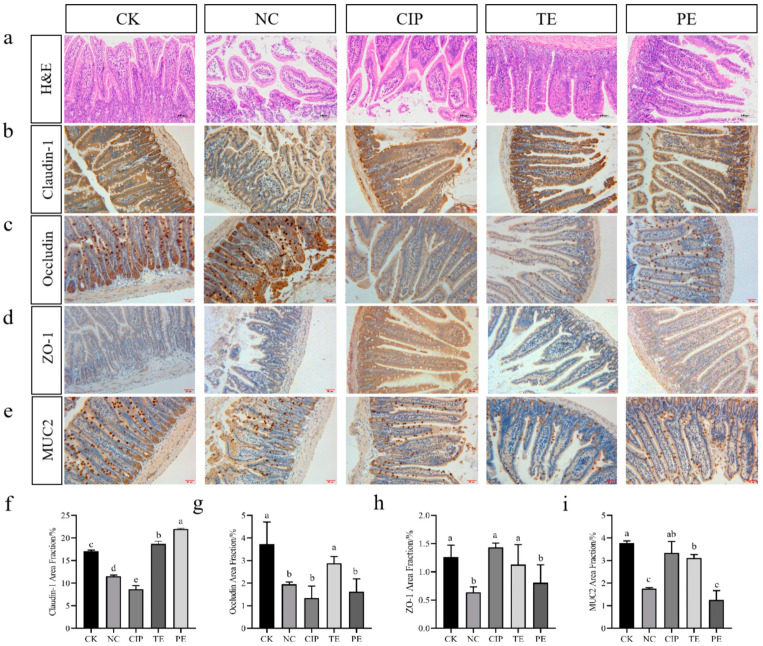
Effects of *L. agilis* SNF7 on the intestinal physical barrier of *E. coli* K99-induced diarrhea mice. (**a**) H&E staining of mouse jejunal tissue (bar = 100 µm); (**b**) expression of Claudin-1 in mouse jejunal tissue (bar = 100 µm); (**c**) expression of occludin in mouse jejunal tissue (bar = 100 µm); (**d**) expression of ZO-1 in mouse jejunal tissue (bar = 100 µm); (**e**) expression of MUC2 in mouse jejunal tissue (bar = 100 µm); (**f**) area fraction of Claudin-1; (**g**) area fraction of occludin; (**h**) area fraction of ZO-1; (**i**) area fraction of MUC2. The same letters on the column show the difference is not significant (*p* > 0.05). The difference is significant (*p* < 0.05) when there are no identical letters between the columns.

The experimental groups’ jejunum tissues stained with H&E showed clear structural changes (Figure 4a). The intestinal villi, which were made up of goblet cells and columnar epithelial cells, were quantitatively plentiful and the mucosal layer appeared structurally intact in the CK group. Compared with the CK group, the NC group exhibited significant damage. Following the administration of *E. coli* K99, certain intestinal villi were fragmented, mucosal epithelial cells exhibited hydropic degeneration, and the intestinal villous epithelium separated from the lamina propria. However, compared with the NC group, the intervention of *L. agilis* SNF7 and ciprofloxacin alleviated the morphological damage to the jejunal mucosa, decreased the number of intestinal villi epithelial cells, and decreased the degree of mucosal epithelial cell infiltration.

Following treatment with *E. coli* K99, each group’s animals showed a decrease in TJ protein and MUC2 expression levels (*p* < 0.05; Figure 4b–e). To varied degrees, the treatment of ciprofloxacin and *L. agilis* SNF7 reversed these decreases in the expression levels of claudin-1, occludin, ZO-1, and MUC2 (Figure 4f–i). Claudin-1, occludin, ZO-1, and MUC2 were all expressed at higher levels in the TE group than in the NC group, and claudin-1 was even more highly expressed than in the CK group (*p* < 0.05). In contrast to the NC group, the inhibition of *L. agilis* SNF7 only resulted in an increase in claudin-1 expression and did not substantially reverse the changes in occludin, ZO-1, and MUC2 expression. Similarly, the CIP group did not significantly restore the expression of occludin and claudin-1, although they did increase the expressions of ZO-1 and MUC2. In comparison to the animals in the PE group, the mice in the TE group exhibited an effectively enhanced barrier function overall.

### 2.12. Effects of L. agilis SNF7 on E. coli-Induced Inflammatory Factor Secretion in Mice Jejunum Tissues

**Figure 5:**
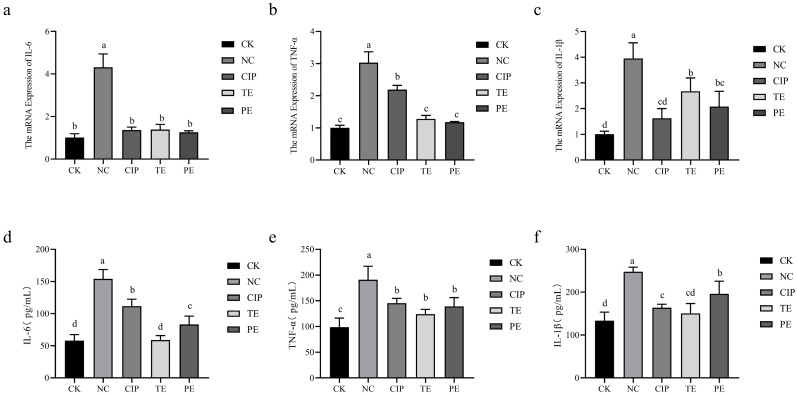
Effects of *L. agilis* SNF7 on *E. coli*-induced inflammatory factor secretion in mice jejunum tissues. (**a**) Relative expression of IL-6 mRNA; (**b**) relative expression of TNF-α mRNA; (**c**) relative expression of IL-1β mRNA; (**d**) IL-6 content; (**e**) TNF-α content; (**f**) IL-1β content. The same letters on the column show the difference is not significant (*p* > 0.05). The difference is significant (*p* < 0.05) when there are no identical letters between the columns.

The mRNA levels of IL-6, TNF-α, and IL-1β were clearly higher in mice treated with NC than in the CK group (*p* < 0.05; Figure 5a–c). Conversely, the TE and PE groups’ *L. agilis* SNF7 intervention decreased the levels of IL-6, TNF-α, and IL-1β in mice treated with *E. coli* K99 (*p* < 0.05), while the mRNA levels of IL-1β were higher than those in the CK group (*p* < 0.05). At the same time, ciprofloxacin intervention decreased IL-6, TNF-α, and IL-1β levels in mice treated with *E. coli* K99 (*p* < 0.05); however, TNF-α mRNA levels were higher than those in the CK group (*p* < 0.05).

ELISA was used to identify the release of inflammatory factors IL-6, TNF-α, and IL-1β in the jejunum tissues of mice infected with *E. coli* K99 (Figure 5d–f). The NC group had considerably higher levels of IL-6, TNF-α, and IL-1β than the CK group (*p* < 0.05). In comparison to the NC group, the CIP, TE, and PE groups had significantly lower levels of inflammatory factors (IL-6, TNF-α, and IL-1β) (*p* < 0.05).

### 2.13. Effect of L. agilis SNF7 on NF-κB and MAPK Signaling Pathways in E. coli K99-Induced Diarrhea Mice

**Figure 6:**
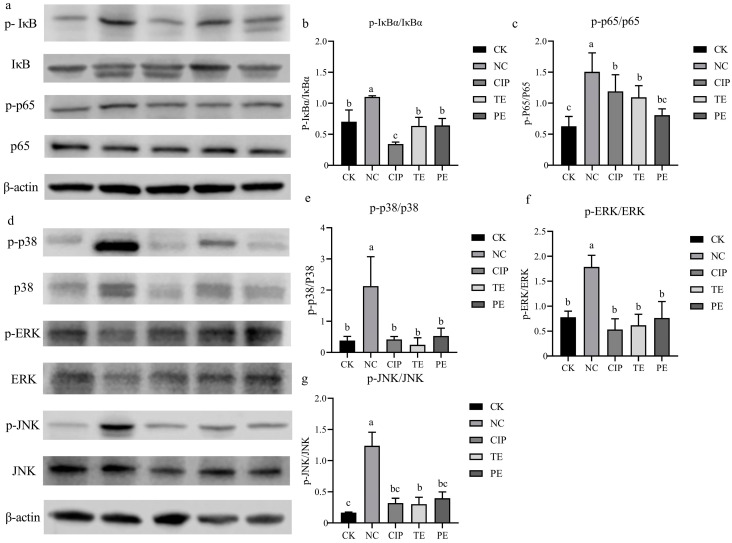
Effect of *L. agilis* SNF7 on NF-κB and MAPK signaling pathways in *E. coli* K99-induced diarrhea mice. (**a**) Protein bands of NF-κB signaling pathways; (**b**) p-IκBα/IκBα; (**c**) p-p65/p-65; (**d**) protein bands of MAPK signaling pathways; (**e**) p-p38/p38; (**f**) p-ERK/ERK; (**g**) p-JNK/JNK. The same letters on the column show the difference is not significant (*p* > 0.05). The difference is significant (*p* < 0.05) when there are no identical letters between the columns.

The ratios of p-IκB/IκB, p-p65/p65, p-p38/p38, p-JNK/JNK, and p-ERK/ERK were significantly increased in the NC group (*p* < 0.05) in comparison to the mice in the CK group. In contrast to the NC group, the CIP, TE, and PE groups displayed significantly lower ratios of p-p-IκB/IκB, p-p65/p65, p-p38/p38, p-JNK/JNK, and p-ERK/ERK (Figure 6).

### 2.14. Modulation Effect of L. agilis SNF7 on the Gut Microbiota in E. coli K99-Induced Diarrhea Mice

**Figure 7:**
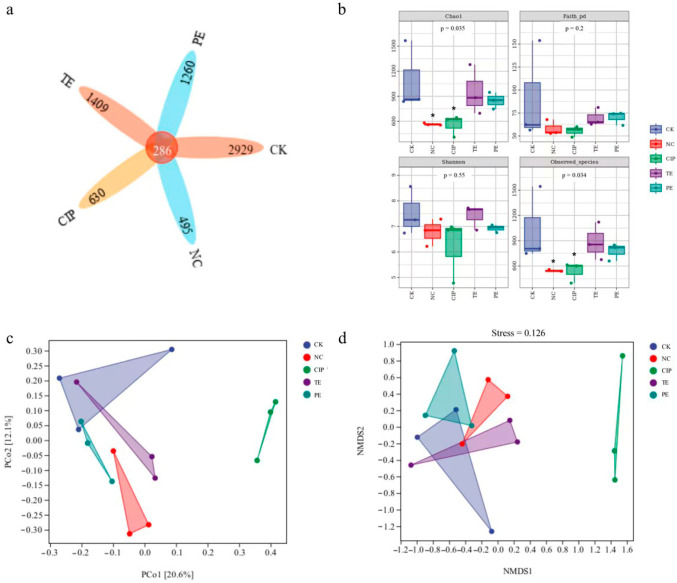
Venn diagram, α diversity analysis, and β diversity analysis of cecum microorganisms in each group. (**a**) OTU Wayne diagram; (**b**) α diversity analysis; (**c**) beta-diversity analysis: PCoA analysis; (**d**) beta-diversity analysis: NMDS analysis. * Compared with the CK group, * means significant difference (*p* < 0.05).

With 286 overlapping microorganisms found among the five groups, the Venn diagram graphically represents the overlay of OTUs among groups, as seen in Figure 7a. The OTUs in the TE and PE groups were more prevalent than those in the NC and CIP groups. Comparative analysis of the α-diversity of the microbial communities in the three groups revealed that the NC and CIP groups had significant suppressions in their richness and diversity, while the *L. agilis* SNF7 group improved these parameters (Figure 7b). Distinct clustering separations between the CK, NC, and CIP groups were shown by the findings of Principal Coordinate Analysis (PCoA) and Non-metric Multidimensional Scaling (NMDS) (Figure 7c,d), indicating different gut microbiota topologies.

**Figure 8:**
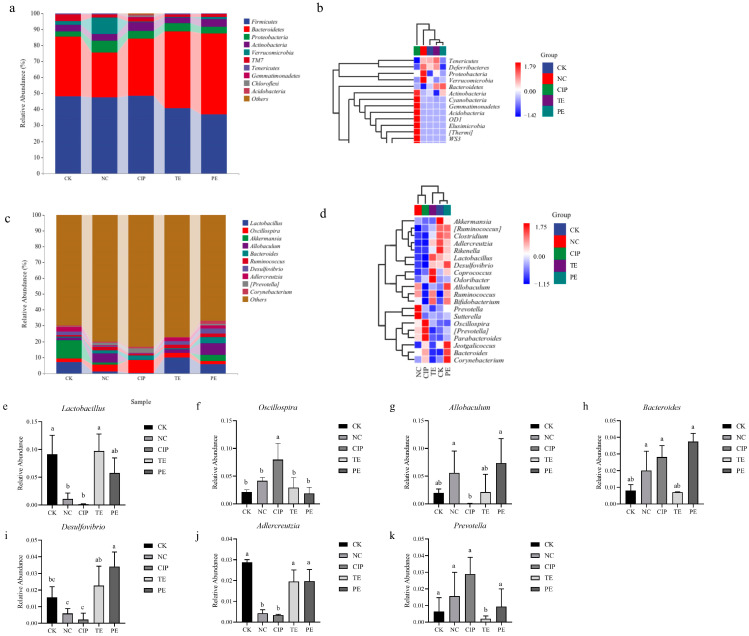
Modulation effect of *L. agilis* SNF7 on the gut microbiota in *E. coli* K99-induced diarrhea mice. (**a**) Phyla-level species distribution histogram; (**b**) phylum-level species composition heat map; (**c**) genus-level species distribution histogram; (**d**) generic-level species composition heat map; (**e**–**k**) relative abundance of genera with genus-level differences between groups. The same letters on the columns show the difference is not significant (*p* > 0.05). The difference is significant (*p* < 0.05) when there are no identical letters between the columns.

We then looked into the changes in gut microbiota compositions brought on by the administration of *L. agilis* SNF7 and *E. coli* K99. *Firmicutes*, *Bacteroidetes*, *Proteobacteria*, *Actinobacteria*, and *Verrucomicrobia* are the most prevalent phyla (Figure 8a,b). At the phylum level, Bacteroidetes and TM7 showed a considerable decline, while Verrucomicrobia and Proteobacteria showed a sharp increase in the NC group relative to the healthy group. The *L. agilis* SNF7 significantly decreased the relative abundance of Proteobacteria and Verrucomicrobia in contrast to the NC group, while increasing the relative abundance of Bacteroidetes and TM7 (Figure 8c,d). At the genus level, the abundances of *Lactobacillus* and *Adlercreutzia* in NC were lower than in the CK group, the phenomenon partly reversed by the TE and PE groups (Figure 8e,j). *Lactobacillus* and *Allobaculum* were undetectable in the CIP group (Figure 8e,g). No substantial difference in the majority of genus (such as *Lactobacillus*, *Akkermansia*, *Oscillospira*, *Allobaculum*, and *Ruminococcus*) among the TE, PE and CK groups was observed; however, *Desulfovibrio* was higher in the PE group compared to the CK group (Figure 8i), and *Prevotella* was lower in the PE group compared to the CK group (Figure 8k).

## 3. Discussion

The gut microbiota, the most intricate and physiologically varied microbial community in the host, has progressively acquired vital functions as a result of co-evolution with the host over thousands of years, thus playing a key role in preserving the health of the host [17,18,19]. Symbiotic microorganisms in the intestinal microbiota resist the colonization of pathogenic microorganisms in the intestinal tract, through the synthesis of bacteriostatic substances, competition for nutrients, and the seizure of adherence sites, thereby preventing the occurrence of intestinal diseases caused by pathogenic bacterial infections [20,21,22,23]. In this study, we evaluated the potential probiotic properties and performed a genome-wide analysis of *L. agilis* SNF7 isolated from the feces of healthy calves. Furthermore, our investigation revealed that *L. agilis* SNF7 has the ability to alleviate diarrhea caused by *E. coli*.

To evaluate a possible probiotic candidate, it is necessary to consider a strain’s capacity to inhibit the growth of infections, tolerance to the gastrointestinal environment, ability to multiply rapidly, and harmlessness to the host. The culture supernatant of *L. agilis* SNF7 exhibited strong bacteriostatic activity against *E. coli* O_111_:K_58_ (B_4_), *S. aureus*, and *E. coli* K99 in vitro, which may be attributed to the secretion of organic acids and hydrogen peroxide by *L. agilis* SNF7. This characteristic might provide the chosen probiotic strains the edge over their rivals in the host gut. The survival of probiotic bacteria through the gastrointestinal tract is crucial to exert a positive effect when administered to animals. Some strains of LAB, including *Lactobacilli* and *Lactococcus*, have high survival rates in gastrointestinal conditions [24,25]. Likewise, *L. agilis* SNF7 remained viable in extremely acidic or bile salt concentration conditions; it was able to withstand up to 61.58% and 68.62% in artificial gastric and intestinal fluids, respectively. Additionally, in suitable conditions, *L. agilis* SNF7 can rapidly reach a logarithmic growth period for reproduction and cause a rapid decrease in environmental pH to inhibit the growth of pathogenic microorganisms. Hemolysis and antimicrobial susceptibility are two crucial indicators in the probiotics’ harmlessness evaluation [26]. Not only is *L. agilis* SNF7 susceptible to a broad spectrum of antibiotics, but also it is not hemolytic, fulfilling one of the criteria used to select probiotic bacteria. *L. agilis* SNF7 presented antibiotic resistance to kanamycin, which might be an advantage considering a joint use for probiotic therapy in the future. Given these in vitro characteristics from our investigation, which aligns with the criteria used in previous studies to assess potential probiotic candidates [27,28], we believe that *L. agilis* SNF7 represents a viable substitute for antibiotics in the clinical treatment of *E. coli* line diarrhea.

A thorough assessment of the strain is necessary before an isolated probiotic for use in veterinary production, and this assessment is based on the complete genome sequence. Probiotics have unique health advantages, and whole genome sequencing is useful for a deeper investigation of the protective mechanisms of various strains. Based on the whole genome sequence of *L. agilis* SNF7, we discovered that *L. agilis* SNF7 has a strong capacity for utilizing carbohydrates, which serves as a foundation for the strain’s ability to survive in a variety of conditions. Furthermore, genes linked to antibacterial substance production have been discovered in *L. agilis* SNF7, which may prevent the growth of pathogenic microbes or even kill them. In addition to bacteriostatic substances, *L. agilis* SNF7 also contains genes encoding a variety of metabolites, including ascorbic acid, terpenoids, nicotinic acid and nicotinamide, secondary bile acids, taurine and hypotaurine, etc. Nicotinamide, in particular, is the primary precursor of nicotinamide adenine dinucleotide (NAD^+^), which activates Sirtuin 1 (SIRT1), a protein that inhibits the NF-κB signaling pathway and reduces the release of inflammatory factors [29,30]. In the intestinal tract, secondary bile acids possess antibacterial properties that can prevent the growth of pathogenic microorganisms and preserve the equilibrium of gut flora [31], which is consistent with our earlier findings about the antibacterial activity of *L. agilis* SNF7 in vitro. In a word, *L. agilis* SNF7 exhibits potent antibacterial, antioxidant, anti-inflammatory, and immunomodulatory properties by the production of the aforementioned many metabolites. Apart from metabolite synthesis, 18 bacterial chemotaxis-related genes were annotated in the *L. agilis* SNF7 genome. *L. agilis* SNF7 has an advantage in gut colonization due to its motility and chemotaxis. While bacterial chemotaxis does not possess an antibacterial property, it may impact the localization and survival of bacteria within the host by controlling bacterial movement and distribution, thereby resisting the pathogenic microbial colonization of the host [32,33]. Lactic acid bacteria have been shown to interact with host cell sugar molecules by expressing certain surface proteins that improve their adherence [23]. Through a mechanism of competitive exclusion, this adhesion not only aids in the colonization of *lactobacilli* in the intestine, but also inhibits the adherence of harmful bacteria. Although *L. agilis* SNF7’s adhesion capabilities were not assessed in our work, it is possible that it may adhere to host cells efficiently based on the examination of its genomic sequence. *L. agilis* SNF7 may be able to colonize the intestine and contribute to the prevention of infection and pathogen adherence thanks to this adhesion ability. The discovery of the CRISPR-Cas system in the genome of *L. agilis* SNF7 implies that the host genome’s immunological self-defense systems can defend against intrusive foreign substances [34]. The CARD database, an integrated database of genes linked to antibiotic resistance, is a valuable resource for researching antibiotic resistance in human, animal, and environmental flora, as well as for deciphering the mechanisms underlying antibiotic resistance [35]. The CARD database predicted the aminoglycoside resistance gene *APH (7″)-Ia* and the macrolide resistance gene *mefA* in the *L. agilis* SNF7 genome. Additionally, *L. agilis* SNF7 exhibits resistance to aminoglycosides and macrolides via antibiotic inactivation and antibiotic efflux pumps, respectively. A possible advantage for *L. agilis* SNF7’s use in the probiotic industry is that its genome-wide content of resistance genes is generally low. Additionally, the existence of these two resistance genes opens the door to the possibility of using *L. agilis* SNF7 when combined with antibiotics.

*E. coli* is the main cause of diarrhea in newborn animals, resulting in dehydration, diarrhea, and even death in affected animals. Antibiotic overuse in the treatment of *E. coli*-induced diarrhea results in drug residues and the emergence of pathogen resistance. According to a study, *E. coli* isolates from Spain’s diarrhea samples were resistant to over four different antibiotics [36]. Diarrheagenic *Escherichia coli* showed 38% resistance to 10 antibiotics in Hohhot, China [37]. Because of their potential to help avoid harmful infections, probiotics have attracted a lot of interest. Lactic acid bacteria can be given to calves at an early age of their growth to help with feed efficiency, daily weight gain, and diarrhea incidence [38]. The intestinal damage and diarrhea brought on by *E. coli* K99 were considerably reduced by multispecies probiotics (*Saccharomyces cerevisiae*, *Lactobacillus acidophilus* S5, and *Bacillus subtilis*) [38]. Therefore, in order to investigate the protective effects of *L. agilis* SNF7 and clarify its mechanism of action, our study created an *E. coli* K99-infected mouse model. After a week of gavage with *E. coli* K99, the experimental group of mice showed symptoms such as diarrhea, weight loss, liver shrinkage, and splenomegaly, demonstrating that the *E. coli* K99 infection model was successfully established compared to the control group. The diarrhea, weight loss, and organ index abnormality values were all reduced in mice in the TE and PE groups that received *L. agilis* SNF7 supplements. Further evidence of *L. agilis* SNF7’s effectiveness in reducing infection symptoms in mice was provided by the decline in the fecal score. These findings are consistent with Wang et al.’s study, which found that *Lactobacillus casei* LC2W increased food intake and significantly decreased the rates of diarrhea and weight loss in infected mice [39].

The intestinal barrier is an essential component of the body that is responsible for both digestion and nutrition absorption. It also serves as the body’s first line of defense against external pathogens [40]. The intestinal epithelial microvilli and mucosal lining were damaged in our study, indicating that *E. coli* K99 compromised the integrity of the colon barrier in mice. On the other hand, *L. agilis* SNF7-treated mice demonstrated considerably improved colon tissue pathology, less severe inflammatory infiltration, better-preserved intestinal epithelial structures, and reduced intestinal damage, supporting the findings of Wu et al. [41]. Maintaining the integrity of the intestinal barrier depends on tight junctions (TJs), a type of intercellular connection made up of structural proteins, such Claudin, occludin, Zos, and so on [39]. Infection with *E. coli* K99 markedly decreased ZO-1, occludin, and Claudin-1 expression. On the other hand, treatment with *L. agilis* SNF7 successfully reversed these alterations. This is consistent with previous studies that revealed *Lactobacillus salivarius* can counteract the colon’s decreased TJ protein expression [42]. Mucin is secreted by goblet cells in the intestinal tissues. When mucin is absent, pathogenic microbes can directly contact the intestinal epithelial cells in the organism, which can result in intestinal inflammation brought on by bacterial infections [43]. Our experiment revealed that the supplementation of mice with *L. agilis* SNF7 prevented an *E. coli* K99-induced decrease in MUC2 expression. Consistent with our findings, the intervention of *Lactobacillus paracasei* might promote the number of goblet cells and MUC2 expression in the intestine, hence maintaining the integrity of the colonic mucosal layer [44]. Thus, we postulated that, by upregulating the expression of tight junction proteins and mucins, *L. agilis* SNF7 may enhance the integrity of the mouse intestinal barrier against pathogenic bacterial infection.

Proinflammatory cytokines play an important role in the development of intestinal inflammatory diseases caused by pathogenic microorganisms [45]. Consistent with our results, *E. coli* invasion of the organism can result in an increased expression of pro-inflammatory cytokines in animal tissues, including IL-1β, IL-6, and TNF-α [46]. Notably, the treatment with *L. agilis* SNF7 prevented these elevations in the concentrations of IL-1β, IL-6, and TNF-α in the colon tissue. The findings show that the number of cytokines produced by the intestinal epithelial cells after infection with ETEC is significantly reduced when *Lactobacillus plantarum* was pretreated [47]. The NF-κB signaling pathway, which is one of the most-studied signaling pathways in inflammatory responses, regulates the immune response by modulating gene expression involved in immune and inflammatory responses [48]. As a crucial signal transduction pathway in the cytoplasm, the MAPK signaling pathway is involved in many different cellular processes, including immune defenses, apoptotic responses, cellular inflammation, and stress, which is especially crucial for epithelial cellular immunity and plays a key role in controlling inflammatory processes [49]. In this study, the activation of the NF-κB and MAPK signaling pathways was observed in the mouse intestinal tissues infected with *E. coli* K99. However, the activation of the NF-κB and MAPK signaling pathways was inhibited by *L. agilis* SNF7 intervention. Similarly, by attenuating the phosphorylation of p38 MAPK and blocking the NF-κB signaling pathways, *Lactobacillus salivarius* lowers the expression of cytokines linked to inflammation [50]. *Lactobacillus plantarum* inhibits the activation of NF-κB and p38 MAPK signaling pathways through unique metabolites, hence reducing the production of pro-inflammatory cytokines [9]. Our comprehensive understanding of *L. agilis* SNF7’s antidiarrheal actions can be further enhanced by considering the possibility that the NF-κB and MAPKs signaling pathways are one of the molecular protective mechanisms that shield mice from *E. coli* K99-induced diarrhea.

Given the diverse and critical role of the gut microbiota in animal health, disruptions in microbial composition may have far-reaching detrimental consequences on the body’s immune system, leading to the development of different chronic inflammatory disorders [51]. The use of antibiotics may therefore worsen the dysbiosis of the gut microbiota, increasing the quantity of pathogenic bacteria that can cause a range of dangerous bacterial infections in humans and animals, ultimately resulting in the development of illnesses [52,53]. Probiotics can protect the host by altering the makeup and metabolism of the intestinal flora by preventing the growth and reproduction of pathogenic microorganisms in the gut [54]. In this investigation, we discovered that *L. agilis* SNF7 effectively prevented the *E. coli* K99 infection-induced reduction in microbial diversity in the mice’s gut microbiota. However, the general similarity between the microbial community structure of the mouse cecum and that of the control group was significantly altered by ciprofloxacin therapy. Changes in the quantity of specific bacteria within the intestinal microbial community frequently result in modifications to the organism’s health condition. For instance, the abundance of *Actinobacteriota*, *Bacteroides*, and *Proteobacteria* is a key indicator of the health of the human gut microbiota [55,56,57]. At the phylum level, a decrease in the relative abundance of the *Actinobacteriota* and *Bacteroides* and an increase in the relative abundance of the *Brevibacteria* phylum was observed following *E. coli* K99 infection, while *L. agilis* SNF7 administration prevented these modifications. *L. agilis* SNF7 also raised the relative abundance of *Lactobacillus* and *Adlercreutzia*. Notably, ciprofloxacin treatment led to highly significant decreases in the abundance of numerous beneficial intestinal bacteria. These observations align with the findings of Ran et al. *Enterococcus faecalis* protects against *Salmonella* infection by adjusting the abundance of gut microorganisms and their metabolic pathways [58]. The results show that *L. johnsonii*, *L. plantarum*, and *L. rhamnosus* elevate the relative abundance of gut microorganisms in the intestine of EHEC-infected mice [59].

According to our findings, *L. agilis* SNF7 is a probiotic that shows promise in the treatment and prevention of *E. coli*-induced diarrhea. However, more research is still needed to fully understand *L. agilis* SNF7’s long-term stability and colonization capacity in vivo. Furthermore, it is urgently necessary to investigate whether *L. agilis* SNF7 plays a role in transferring antibiotic resistance to other microorganisms in the microbiota.

## 4. Materials and Methods

### 4.1. Isolation and Identification of Probiotics in Calf Feces

The fecal samples were serially diluted and inoculated with De Man Rogosa and Sharpe agar (MRS; AOBOX, Beijing, China) after being resuspended in 9 mL of saline solution (0.9% [*w*/*v*] NaCl). Colonies were streaked on MRS agar plates based on morphological differences after 48 h at 37 °C. This process was repeated two or three times until a single pure colony of the same form was achieved. All bacterial isolates were categorized using Gram staining and microscopic analysis.

Following the manufacturer’s instructions, a DNA extraction kit (TransGen Biotech, Beijing, China) was used to extract the bacteria’s DNA, and a Nanodrop instrument (Nanodrop 2000 Thermo Fisher Scientific, Waltham, MA, USA) was used to measure the concentration. The 16S rRNA gene was amplified from the strain’s genomic DNA using the universal primers 27F (5′-AGA GTT TGA TCC TGG CTC AG-3′) and 1492R (5′-CTA CGG CTA CCT TGT TAC GA-3′) in a polymerase chain reaction (PCR). The thermal cycling settings were 94 °C for 5 min, followed by 35 cycles of denaturation at 94 °C for 30 s, primer annealing at 55 °C for 45 s, elongation at 72 °C for 90 s, and thermal retardation at 72 °C for 10 min. Additionally, Sangon Biotech Company (Shanghai, China) sequenced the PCR products, and the NCBI website’s nucleotide BLAST was used for analysis.

### 4.2. Strain Survival in the In Vitro-Simulated Gastrointestinal Conditions

We evaluated the probiotic bacteria’s ability to survive in vitro-mimicked gastrointestinal conditions. After being in MRS broth for 24 h at 37 °C, the bacterial isolates were centrifuged (4000× *g*/10 min). When the final cell count reached 10^8^ CFU/mL, the resultant pellet was resuspended in MRS broth after being cleaned with phosphate buffered saline (PBS) buffer (20 mM, pH 7.2). Simulated intestinal fluid (the MRS broth added with 1 mg/mL of trypsin and pH 8.0), simulated gastric fluid (the MRS broth supplied with 1 mg/mL of pepsin and pH 3.0), and bile salt solution were all administered to the bacteria. The MRS broth served as a control for the bacteria. In short, 4750 μL of MRS broth and 250 μL of cell resuspension solution were mixed, and the mixture was incubated for 4 h at 37 °C. Following the measurement of the culture’s optical density at a 600 nm wavelength (OD600) using a spectrophotometer (DeNovix, Wilmington, DE, USA), survival rates (SRs) were calculated as follows:

SR = A_t_/A_c_ × 100%

where A_c_ stands for the control group’s OD600 at 4 h and A_t_ for the treatment group’s OD600 at 4 h.

### 4.3. Antibacterial Activity of Probiotic Cell-Free Supernatant

The antibacterial properties of the bacterial strains obtained here were assessed using the well diffusion method. *Staphylococcus aureus* (CVCC186158), *Escherichia coli* K99 strain isolated from a calf’s diarrheal feces, and *Escherichia coli* O_111_:K_58_(B_4_) (CVCC1450) were used to measure the antibacterial activity. After being in MRS broth for 24 h at 37 °C, the bacterial isolates were centrifuged (12,000× *g*/10 min). After that, 200 µL of the supernatant was placed in wells with a diameter of 6 mm on a Mueller–Hinton (MHA) agar plate that had been pre-inoculated with the pathogen bacteria (100 µL, 10^6^ CFU/mL). The control was sterile MRS broth. For 12 h, the inoculated MHA plates were kept in an incubator that was adjusted to 37 °C. The wells’ surrounding zone of inhibition diameters (mm) were measured.

### 4.4. Propertiy of Antibacterial Compounds Produced by the Probiotics Isolates

We determined whether the production of acid or the synthesis of bacteriocin was responsible for the antibacterial activity. To remove any possible acidic effects, 1 mol/mL of NaOH was used to bring the supernatant’s pH down to 7.0. Following a one-hour incubation period at 37 °C, the supernatant was subsequently treated separately with proteinase K (30 U/mg, 5 mg/mL) and catalase (5220 U/mg, 1 mg/mL). The well diffusion testing method was used to evaluate the supernatant’s efficacy against harmful microorganisms. The experiment’s control was the supernatant that had not been treated with any enzymes.

### 4.5. Antibiotic Susceptibility of the Probiotic Isolates

Antimicrobial susceptibilities of the isolated and identified bacterial against different antibiotics, including penicillin G (10 Units), ampicillin (10 μg), tetracycline (30 μg), cefatriaxone (30 µg), erythromycin (15 µg), clindamycin (2 μg), ciprofloxacin (5 μg), kanamycin (30 µg), and chloramphenicol (30 µg) were tested by the Kirby–Bauer Disc-Diffusion method according to the recommendation of the Clinical and Laboratory Standards Institute. The bacterial suspension, at 10^9^ CFU/mL, was inoculated by swabbing on MRS agar. After drying, the standard antibiotic discs were placed on the surface of the agar. After incubation, the antibiotic susceptibilities of probiotic isolates were determined according to the values of inhibition zones. Antibiotic sensitivity was categorized as R, IR, or S. The inhibition zones were measured in millimeters, with zones larger than 20 mm considered sensitive and zones smaller than 14 mm considered resistant.

### 4.6. Hemolytic Activity of the Probiotic Isolates

Using a blood agar media, the lysis of sheep red blood cells by bacteria was examined. Spots on trypticase soy agar containing 5% (*v*/*v*) defibrinated sheep blood were used to inoculate the isolated organisms. A clean zone surrounding the colonies was then inspected after the plates had been incubated for 24 h at 37 °C. Positive hemolytic activity was defined as the development of a clear or semitransparent zone surrounding the site.

### 4.7. Growth and Acid Production Curves of the Probiotic Isolates

The bacterial isolates were subjected to MRS broth at 37 °C for 24 h. A spectrophotometer (DeNovix, USA) was then used to measure the OD600 values every two hours for 24 h after the suspension had been cultured in MRS broth (1%, *v*/*v*) at 37 °C. A FiveEasy standard pH meter (Mettler Toledo, Columbus, OH, USA) was used to monitor the bacterial culture’s pH every two hours until it was twenty-four hours along. The average value from three consecutive samples was used to plot the strain’s growth and acid production curves.

### 4.8. Genome Sequencing, Assembly, and Bioinformatic Analyses

Following the manufacturer’s instructions, genomic DNA was extracted using the EasyPure^®^ Bacteria Genomic DNA Kit (TransGen Biotech, Beijing, China). A Nanodrop ND2000 spectrophotometer (ThermoFisher Scientific, Waltham, MA, USA) was used to assess the concentration and purity of the collected DNA. Following the usual procedure supplied by Oxford Nanopore Technologies (ONT), the library was created using the SQK-LSK109 ligation kit (Oxford Nanopore Technologies, Oxford, UK) and measured using a Qubit fluorometer (Thermo Fisher Scientific, Waltham, MA, USA). Whole genome sequencing was carried out using the Ion PromethION48 System.

Canu v1.5 software was used to assemble the acquired reads for genome assembly, and circlator v1.5.5 was then used to cycle the assembly genome. Prodigal v2.6.3 was used for coding gene prediction in order to predict genomic components. Infernal v1.1.3 was used to predict ribosome RNA (rRNA) genes, whereas tRNAscan-SE v2.0 was used to predict transfer RNA (tRNA) genes. RepeatMasker was used to predict repetitive sequences. Using AntiSMASH v5.0.0, secondary metabolic gene clusters were predicted.

The predicted proteins were blasted (e-value: 1 × 10^−5^) against KEGG and eggNOG for functional annotations. The GO annotation was performed using Blast2go v2.5. Additionally, by blasting against the CAZy, TCDB, CARD, and PHI databases, the pathogenicity and drug resistance could be investigated.

### 4.9. Animal Experiments

Since Kunming mice have a comparatively robust immune system and are well-adaptable to lab settings, we decided to utilize them to create a diarrhea model. From Specific Pathogen Free (SPF) Biotechnology Co. (Beijing, China), thirty Kunming mice (15 males and 15 females; weight: 28–32 g; age: 4–6 weeks old) were acquired. The mice were kept at 20 °C ± 2 °C with a 12 h light and 12 h dark cycle, kept at 45% ± 10% humidity, and provided unlimited access to sterile food and water while being grown in a pathogen-free environment. Three mice were housed in each cage according to uniform sex and in the same group. The mice were randomly divided into five groups (*n* = 6; 3 males and 3 females) following a week of acclimatization.

Prior to the experiment, *L. agilis* SNF7 was static cultured for 24 h in a centrifuge tube with 10 milliliters of MRS broth medium. To keep out outside oxygen throughout the incubation time, the centrifuge tube was sealed with a sealing film. *E. coli* was inoculated with suspension culture in a centrifuge tube with 10 mL of LB broth medium for 12 h (220 r/min). It is important to note that, prior to gavage, the concentration of bacteria extracted from the medium was adjusted using saline. *E. coli* and *L. agilis* SNF7 concentrations were adjusted to 1 × 10^8^ CFU/mL and 1 × 10^9^ CFU/mL, respectively.

The following treatments were administered to the mice in several groups: The control group (CK) received NS orally from days 1 through 14. The mice in the model group (NC) received 0.2 mL of 1 × 10^8^ CFU/mL *E. coli* K99 from days 1 through 7 and 0.9% physiological saline (NS) orally from days 8 through 14. On days 1 through 7, the mice in the ciprofloxacin treatment group (CIP) received 0.2 mL of 1 × 10^8^ CFU/mL *E. coli* K99, and on days 8 through 14, they were administered 65 mg/kg of ciprofloxacin orally. On days 1 through 7, the mice in the treatment group (TE) received 0.2 mL of 1 × 10^8^ CFU/mL *E. coli* K99, and on days 8 through 14, they received 0.2 mL of 1 × 10^9^ CFU/m *L. agilis* SNF7 orally. The mice in the preventive group (PE) received 0.2 mL of *L. agilis* SNF7 (1 × 10^9^ CFU/mL) on days 1 through 7 and 0.2 mL of *E. coli* K99 (1 × 10^8^ 16S rRNA Analysi) orally on days 8 through 14 (Figure 1a).

Days 0, 2, 4, 6, 8, 10, 12, and 14 were used to measure the mice’s body weight. Days 0, 3, 7, 11, and 14 were used to count the number of mice in each group who had diarrhea. Diarrhea rate was estimated as diarrhea mice/total mice in each group times 100%. After administering the serum, the small intestine and cecum contents of each mouse were collected and stored at −80 °C; they were slaughtered on day 15 after blood collection. The livers and spleen were then weighed.

### 4.10. Pathological Analysis

The tissue samples from the jejunum were preserved in a 4% paraformaldehyde fix solution. The intestinal tissues that were fixed were then prepared, cut, and embedded in paraffin. Sections that were 5 μm thick were dewaxed by immersing them in xylene and ethanol after being baked for one hour at 60 °C. Hematoxylin and eosin (HE) staining was applied to the sections. The sections of dyed tissue were examined closely using a light microscope (NIKON, Tokyo, Japan).

### 4.11. Immunohistochemical Analysis

In order to suppress endogenous peroxidase activity, a 3% hydrogen peroxide solution (Sinopharm, Beijing, China) was applied to the jejunum sections after they had undergone antigen repair for the immunohistochemical examination. After 30 min of room-temperature sealing with 3% BSA (Servicebio Technology, Wuhan, China), the tissues were incubated with a primary antibody for the entire night at 4 °C. The sections were examined under a microscope after being incubated with a secondary antibody and stained with hematoxylin and DAB (Servicebio Technology, Wuhan, China).

### 4.12. Enzyme-Linked Immunosorbent Assay

After weighing the jejunum tissue, precooled PBS (0.01 M, pH = 7.4) was added at a ratio of 1:9 (*w*/*v*) to create a 10% tissue homogenate. After centrifuging the homogenate for 10 min at 4 °C and 12,000 rpm, the supernatants were transferred into sterile tubes. ELISA kits (mlbio, Shanghai, China) were used to assess the levels of IL-1β, IL-6, and TNF-α. Other procedures were then carried out in accordance with the manufacturer’s instructions.

### 4.13. RNA Extraction and Quantitative Real-Time PCR

A NanoDrop device (Thermo Fisher Science, UK) was used to extract and quantitatively assess the total RNA of uterine tissue. cDNA synthesis was performed using the FastKing-RT SuperMix (Quanshijin, Beijing, China) kit. The SYBR Green qPCR Master Mix (Quanshijin, Beijing, China) was used to simplify the RT-qPCR using the CFX96 Real-Time PCR System (Bio-Rad, Hercules, CA, USA). Using the instrument’s default melting curve acquisition software, the PCR amplification process involved predenaturing at 95 °C for 30 s, 95 °C for 10 s, 60 °C for 30 s, and 40 cycles. Sangon Biotech Co., Ltd. (Shanghai, China) produced the primers. The specific primers were IL-6 (5′-CTTCTTGGGACTGATGCTGGTGAC-3′; 5′-TCTGTTGGGAGTGGTATCCTCTGTG-3′), IL-1β (5′-CCTGGGCTGTCCTGATGAGAG-3′; 5′-TCCACGGGAAAGACACAGGTA-3′), TNF-α (5′-GGACTAGCCAGGAGGGAGAACAG-3′; 5′-CAATGTGTCCGTCGTGGATCT-3′), GAPDH (5′-CAATGTGTCCGTCGTGGATCT-3′; 5′-GTCCTCAGTGTAGCCCAAGATG-3′). Gene expression levels were normalized to the GADPH gene, employing the 2^−∆∆Ct^ method.

### 4.14. 16S rRNA Analysis of the Contents of the Cecum

The V3–V4 region of the bacterial 16S rRNA gene was amplified after total genomic DNA was isolated from the colon content sample. Following purification, PCR products were labeled to facilitate the library assembly. The Illumina NovaSeq platform (Paisano Bio Ltd., Shanghai, China) was used to sequence the qualified libraries. Alpha diversity (Chao1 and Shannon indices) and beta diversity studies were performed after obtaining high-quality sequences.

### 4.15. Statistical Analysis

Standard error (SEM) or mean ± standard deviation (SD) was used to express the data. Using SPSS (V version 21.0), one-way analysis of variance (ANOVA) was used to assess significant differences (* *p* < 0.05, ** *p* < 0.01). GraphPad Prism 8.0 was used for the data’s graphical representation.

## 5. Conclusions

*L. agilis* SNF7 showed bacteriostatic ability against three pathogenic strains of *E. coli*, *S. aureus*, and *E. coli* K99 in vitro. It also demonstrated good tolerance to the gastrointestinal environment, sensitivity to the majority of antibiotics without hemolysis, good growth and reproduction properties, and the ability to produce acid. Genome analysis results indicate that *L. agilis* SNF7 shows the ability to utilize carbohydrates, amino acids, and glycosyltransferase function. Notably, the *L. agilis* SNF7 genome also has a large number of pathway genes linked to immunomodulation, anti-inflammation, bacteriostasis, and antioxidants. In addition, the *L. agilis* SNF7 strain showed a low genome-wide content of drug resistance genes. These characteristics make it a good candidate for probiotic applications. Our research demonstrated the potential protective effects of *L. agilis* SNF7 against *E. coli* K99 infection in mice. *L. agilis* SNF7 prevented *the E. coli* K99-induced weakening of the gut barrier and preserved the abundance of beneficial microbes. Moreover, it inhibited the increase in the concentration of pro-inflammatory cytokines by inhibiting the activation of the NF-κB and MAPKs signaling pathways. These insights underscore the potential of *L. agilis* SNF7 as a novel therapeutic agent in preventing and treating infections induced by *E. coli* K99.

## Figures and Tables

**Table 1:** General genome features of *L. agilis* SNF7.

Features	Results	Features	Results
Genome size	2,214,422	5S rRNA	8
GC content	41.63	16S rRNA	8
Number of genes	2156	23S rRNA	8
Total gene length	1,940,892	tRNA	91
Proportion of coding genes	87.65	eggNOG	1853
Mean gene length	900.23	GO	1643
Repeat sequence length	2657	KEGG	1211
Repeat sequence content	0.12	VFDB	0

**Table 2:** *L. agilis* SNF7 genome’s antibacterial and anti-inflammatory pathway and related genes.

No	Pathway ID	Description	Gene Number
1	ko00053	Ascorbate and aldarate metabolism	1
2	ko00121	Secondary bile acid biosynthesis	2
3	ko00130	Ubiquinone and other terpenoid–quinone biosyntheses	4
4	ko00362	Benzoate degradation	3
5	ko00401	Novobiocin biosynthesis	1
6	ko00430	Taurine and hypotaurine metabolism	4
7	ko00521	Streptomycin biosynthesis	4
8	ko00523	Polyketide sugar unit biosynthesis	1
9	ko00525	Acarbose and validamycin biosynthesis	1
10	ko00643	Styrene degradation	2
11	ko00760	Nicotinate and nicotinamide metabolism	8
12	ko00900	Terpenoid backbone biosynthesis	13
13	ko01130	Biosynthesis of antibiotics	116
14	ko02030	Bacterial chemotaxis	18

**Table 3:** CRISPR-Cas proteins and CRISPR consensus repeats in the genome of *L. agilis* SNF7.

Sequence ID	Cas-Type/Subtype	Begin (bp)	End (bp)
136-1	csn2_TypeIIA	148,951	149,622
137-1	cas2_TypeI-II-III	149,619	149,924
138-1	cas1_TypeII	149,902	150,807
139-1	cas9_TypeII	151,005	155,063
1512-1	cas2_TypeIE	1,566,394	1,567,287
1513-1	cas1_TypeIE	1,567,284	1,568,231
1514-1	cas6_TypeIE	1,568,263	1,568,898
1515-1	cas5_TypeIE	1,568,911	1,569,606
1516-1	cas7_TypeIE	1,569,587	1,570,696
1517-1	cse2_TypeIE	1,570,709	1,571,329
1519-1	cas3_TypeI	1,573,397	1,576,135
**Sequence ID**	**Consensus Repeat**	**CRISPR Start (bp)**	**CRISPR End (bp)**
1-1	GTACTAAACATCATTGATTTAACATACTTCTGAGAC	146,511	148,923
2-1	CTAGGCCCCTAATGTGCAAGGAAAATTA	1,009,895	1,009,983
3-1	TGAATCTATTTAACTTAAGAGGAATGTAAAT	1,379,204	1,379,301
4-1	AGGATTACCCCCACTAGTGTGGGGAGAAG	1,565,173	1,566,364

**Table 4:** Prediction of the virulence factor of *L. agilis* SNF7.

Gene_ID	ARO_Name	Resistance Phenotype	Resistance Mechanism
GE000947	*mefA*	macrolides	Antibiotic efflux pump
GE000051	*APH (7″)-Ia*	aminoglycosides	Antibiotic inactivation

## Data Availability

The original contributions presented in the study are included in the article; further inquiries can be directed to the corresponding authors.
